# A Potential New Human Pathogen Belonging to *Helicobacter* Genus, Identified in a Bloodstream Infection

**DOI:** 10.3389/fmicb.2017.02533

**Published:** 2017-12-18

**Authors:** Nathalie L. van der Mee-Marquet, Lucie Bénéjat, Seydina M. Diene, Adrien Lemaignen, Nadia Gaïa, Annemieke Smet, Freddy Haesebrouck, Abdessalam Cherkaoui, Astrid Ducournau, Sabrina Lacomme, Etienne Gontier, Louis Bernard, Francis Mégraud, Alain Goudeau, Philippe Lehours, Patrice François

**Affiliations:** ^1^Service de Bactériologie, Virologie et Hygiène, Hôpital Trousseau, Réseau des Hygiénistes du Centre, CPIAS Centre Val de Loire, Centre Hospitalier Régional Universitaire, and UMR 1282 Infectiologie Santé Publique, Université François-Rabelais, Tours, France; ^2^Laboratoire de Bactériologie, Centre National de Référence des Campylobacters et des Hélicobacters, Bordeaux, France; ^3^Faculté de Médecine et de Pharmacie, URMITE, Aix-Marseille Université, UMR 63, Centre National de la Recherche Scientifique 7278, IRD 198, Institut National de la Santé et de la Recherche Médicale, IHU-Méditerranée Infection, Marseille, France; ^4^Service de Médecine Interne et des Maladies Infectieuses, Centre Hospitalier Régional Universitaire, Hôpital Bretonneau, Tours, France; ^5^Genomic Research Laboratory, University Hospital of Geneva, Geneva, Switzerland; ^6^Laboratory Experimental Medicine and Pediatrics, Faculty of Medicine and Health Sciences, University of Antwerp, Antwerp, Belgium; ^7^Department of Pathology, Bacteriology and Avian Diseases, Faculty of Veterinary Medicine, Ghent University, Merelbeke, Belgium; ^8^Bacteriology Laboratory, Division of Laboratory Medicine, Geneva University Hospitals, Geneva, Switzerland; ^9^Bordeaux Imaging Center, Imagerie Electronique, UMS 3420 Centre National de la Recherche Scientifique US4 Institut National de la Santé et de la Recherche Médicale Université de Bordeaux, Bordeaux, France

**Keywords:** *Helicobacter* sp., human infection, phylogeny, electron microscopy, genome content

## Abstract

We isolated from aerobic and anaerobic blood culture bottles from a febrile patient, a *Helicobacter*-like Gram negative, rod-shaped bacterium that MALDI-TOF MS failed to identify. Blood agar cultures incubated in a microaerobic atmosphere revealed a motile Gram negative rod, which was oxidase, catalase, nitrate reductase, esterase, and alkaline phosphatase positive. It grew at 42°C with no detectable urease activity. Antimicrobial susceptibility testing showed that the organism was susceptible to beta-lactams, gentamicin, erythromycin, and tetracycline but resistant to ciprofloxacin. Electronic microscopy analysis revealed a 3 × 0.5 μm curved rod bacterium harboring two sheathed amphitrichous flagella. Whole genome sequencing revealed a genome 1,708,265 base-pairs long with a GC content of 37.80% and a total of 1,697 coding sequences. The genomic analyses using the nucleotide sequences of the 16S rRNA gene, *hsp60* and *gyrB* genes, as well as the GyrA protein sequence, and the results of Average Nucleotide Identity and *in silico* DNA-DNA hybridization suggest evidence for a novel *Helicobacter* species close to *Helicobacter equorum* and belonging to the group of enterohepatic *Helicobacter* species. As soon as the particular peptide mass fingerprint of this pathogen is added to the spectral databases, MALDI-TOF MS technology will improve its identification from clinical specimens, especially in case of “sterile infection”. We propose to associate the present strain with the Latin name of the place of isolation; Caesarodunum (Tours, France) and suggest “*Helicobacter caesarodunensis*” for further description of this new bacterium.

## Introduction

The *Helicobacter* genus of the *Helicobacteraceae* family is composed of Gram-negative, helical-shaped rods (Euzéby, 1997), distributed into 37 different gastric and non-gastric species, also called enterohepatic Helicobacters. *Helicobacter pylori* is the main species found in human gastric mucosa. It infects a large part of the human population and is associated with chronic gastric inflammation that can evolve toward an ulcer or gastric cancer (such as gastric adenocarcinoma or gastric MALT lymphoma) (Mégraud et al., 2015; Floch et al., 2017). Other Helicobacters from the “*heilmannii* group” can also be detected in human gastric mucosa, probably by cross-contamination from pets or consumption of raw pork meat products (Baele et al., 2009; Ménard et al., 2014). Gastric Helicobacters produce urease to help them survive in the acidic gastric environment. Enterohepatic Helicobacters found in humans are usually associated with diarrheal diseases (such as *Helicobacter pullorum*) (Borges et al., 2015) or systemic infection in immunocompromised hosts (such as *Helicobacter cinaedi*) (Kawamura et al., 2014). They are urease negative and most of them produce a cytolethal distending toxin (CDT) responsible for symptoms of infection such as inflammation (Varon et al., 2014; Péré-Védrenne et al., 2016). Recent data also suggest a potential role for CDT in intestinal carcinogenesis (Ge et al., 2017).

*Helicobacter* species cannot be easily identified when the tools used are based on the study of enzymatic activities, sugar fermentation or assimilation. Today, in clinical laboratories, routine bacterial identification is improved by MALDI-TOF MS technology (Cherkaoui et al., 2011; Angeletti, 2016). Databases associated with MALDI-TOF MS systems are regularly updated with novel spectra in order to increase the accuracy of identification. Nevertheless, this technology fails when the bacterial protein spectrum is absent or too different from those in the database. Fortunately, whole genome sequencing has become popular and easy to access for many clinical laboratories, and many free-access bioinformatics tools can be quickly run to analyze genomic data.

In the present study, we report the identification and characterization of a strain that MALDI-TOF MS failed to identify, belonging to the *Helicobacter* genus, and recovered from a patient suffering a bloodstream infection. Based on the results of routine identification tests, electron microscopy and whole genome sequence analysis, this strain differed from all previously described *Helicobacter* species and was named S15.

## Materials and methods

### Bacterial strain isolation

The S15 strain was isolated from a Bactec blood culture bottle grown aerobically (Becton Dickinson, Le Pont de Claix, France), detected 4 days and 22 h after inoculation. Further culture of strains S15 was performed in trypcase soy agar (Difco, Becton Dickinson) enriched with 5% horse blood for 2–3 days at 37°C or 2 days at 42° under microaerophilic conditions. Plate cultures were incubated in jars using an Anoxomat microprocessor (Mart Microbiology, B.V. Lichtenvoorde, The Netherlands) which creates an atmosphere of 80–90% N_2_, 5–10% CO_2_, and 5–10% H_2_. The strain S15 has been assigned to the CCUG, CIP, and DSMZ collections under the identification numbers 68986, 111406 and 105791, respectively.

### Phenotypic testing

Enzymatic activities were assessed by using the API Campy gallery (bioMérieux, Marcy L'Etoile, France). The presence of catalase and oxidase was investigated. Antibiotic susceptibilities were assessed by the disk diffusion method (+ Etest strip for ciprofloxacin) following the CASFM/EUCAST 2017 recommendations for Campylobacter (in-house Mueller–Hinton (MH) agar supplemented with 5% defibrinated horse blood (MH-F) and 20 mg/L β-NAD; inoculum: McFarland 0.5; incubation during 48 h in a microaerobic environment).

### MALDI-TOF MS assessment

Colonies were subjected to a Bruker Daltonics biotyper device using the Microflex platform (Bruker Daltonics, Bremen, Germany) as previously described (van Veen et al., 2010). Single colonies were transferred onto a target plate and overlaid with matrix solution. FlexControl software (version 3.4) was used for measurements; this version includes eight *Helicobacter* species (*Helicobacter canadensis, Helicobacter canis, Helicobacter cholecystus, H. cinaedi, Helicobacter fenneliae, Helicobacter mustelae, Helicobacter pullorum*, and *H. pylori* with 1–9 spectra for each species). The spectra were analyzed using BioTyper software (version 3.1.66; Bruker) and MBT-Compass and MBT-RUO databases, and extracted profiles were compared to that of *H. pylori*.

### Electronic microscopy

The morphology, cell size, and presence of flagella were determined by transmission electron microscopy. S15 cells were scraped and introduced into a fixative solution of 2.5% glutaraldehyde in 0.1 M cacodylate buffer (pH 7.4) and incubated 1.5 h at room temperature. After centrifugation for 3 min at 5,000 rpm, pellets were mixed/suspended in 500 μl of 0.1 M cacodylate buffer (pH 7.4). A volume of 10 μL of bacterial suspension was adsorbed on carbon grids (Delta Microscopy, Toulouse, France) and negatively stained with freshly prepared 1% (p/v) aqueous uranyl acetate solution. Grids were examined with a transmission electron microscope (H7650, HITACHI, Elexience, France) at 80 kV using high contrast mode, equipped with an Orius 11Mpixel camera (Roper Scientific, Lisses, France).

### Genome sequencing and analysis

Genomic DNA obtained from colonies was purified using the DNeasy kit (Qiagen, Courtaboeuf, France). DNA was subjected to whole genome sequencing on the Illumina HiSeq 2500 (Illumina, San Diego, CA, USA) using 200 base-pair reads with paired-ends according to the Truseq protocol (Illumina), following the manufacturer's recommendations. The quality of sequence reads was assessed with the Fastqc program (http://www.bioinformatics.babraham.ac.uk/projects/fastqc/) and reads were quality filtered using the fastq-mcf program (https://expressionanalysis.github.io/ea-utils/). Genome assembly was performed using the Edena v3 assembler (Hernandez et al., 2014). Assembled genomes were annotated using the Prokka v1.10 program (Seemann, 2014). The “Get_homologues.pl” script was used for core proteome comparisons (Contreras-Moreira and Vinuesa, 2013). The CVTree3 Web Server was used to perform the phylogenetic analysis (Xu and Hao, 2009). Prokka annotation and blastP analysis were performed to identify specific genes involved in the phenotype, evolution, and virulence of the strain. The predicted gene function is based on protein similarity of at least 40% and an E-value of 10e-6. Phylogenetic tree topologies of nucleotide (*16S rRNA* gene, *gyrB* and *hsp60* genes) and predicted amino acid alignments (for GyrA) were constructed with the neighbor-joining method using the MEGA (Molecular Evolutionary Genetics Analysis) software version 6.06 (Trespalacios et al., 2015). The sequences previously created by Ménard et al. were used for that purpose (Ménard et al., 2016) (The accession numbers are provided in Supplemental Table S2); the sequences from strain S15 were obtained after genome sequencing. Four phylogenetic trees were constructed based on the *16S rRNA* gene, *hsp60* and *gyrB* genes and the GyrA protein sequence according to phylogenetic analyses previously described by Ménard et al. (2016). The evolutionary distances were computed using the Kimura 2-parameter method for the *16S rRNA, hsp60* and *gyrB* genes, and the Poisson correction method for GyrA.

### Determination of average nucleotide identity (ANI) and *in silico* DNA-DNA hybridization (DDH)

ANI- and DDH-values were assessed *in silico* using online tools. An assembled genome of strain S15 was uploaded in GGDC (http://ggdc.dsmz.de/ggdc.php#) with the recommended local alignment tool BLAST+ and compared with the closest genomes available in public databases identified in the different phylogenetic trees, to obtain DDH-values. The accession numbers of the strains included in the phylogenetic analyses are provided in Supplemental Table S1. The statistic comparison (logistic regression) used a significant probability value of DDH >79%. Accession numbers of genomes for these comparisons are;: NC_004917.1: *Helicobacter hepaticus* ATCC 51449; NZ_CP014991.1: *H. himalayensis* YS1; NC_020555.1: *H. cinaedi* ATCC BAA-847; NZ_CM000776.2: *H. canadensis* MIT 98-5491; NC_013949.1: *H. mustelae* 12198; NC_014810.2: *Helicobacter felis* ATCC 49179; NZ_CP019645.1: *Helicobacter bilis* AAQJH;, JNOB01000034.1: *H. pullorum* 229313/12 and *Helicobacter equorum* QF1 (EMBL accession number: FZPO00000000). Pairwise ANI-values were obtained using JSpeciesWS (http://jspecies.ribohost.com/jspeciesws/#analyse) with BLAST.

## Results

### Clinical data and bacterial isolation

**Figure 1:**
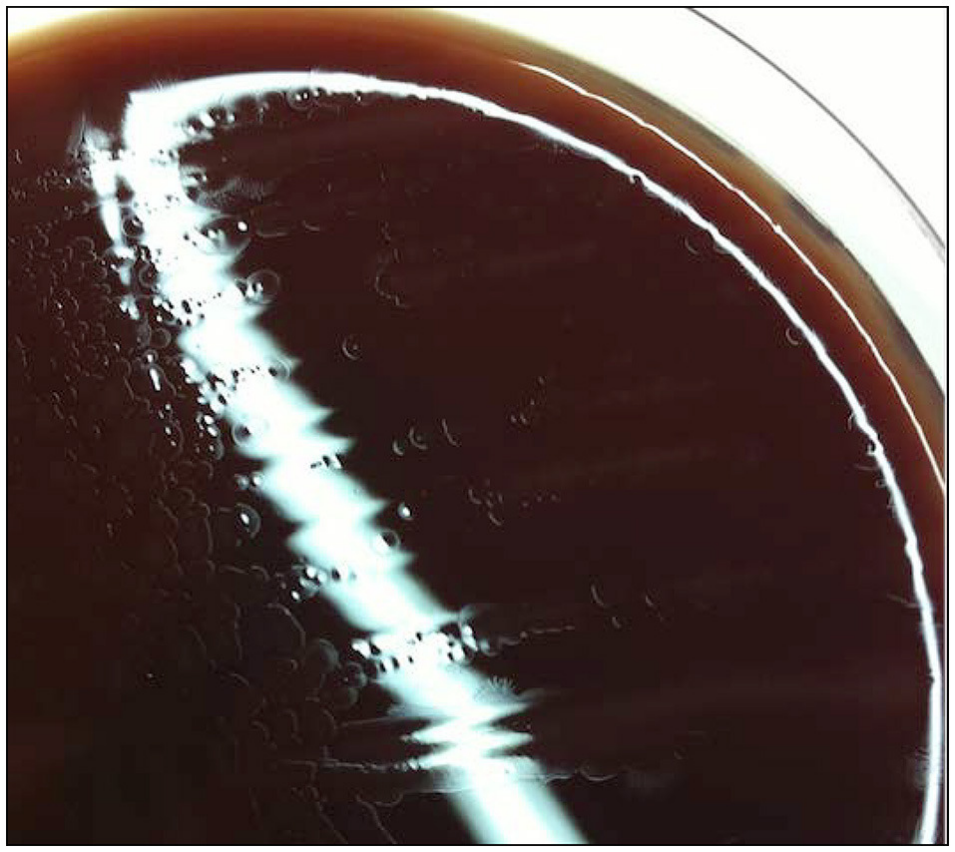
Aspect of S15 colonies on trypcase soy agar. Culture of an identified organism on trypcase soy agar enriched with 5% horse blood for 2–3 days at 37°C.

An 83-year-old man was admitted to the emergency unit complaining of painful ankles. His main medical history included an aortic prosthetic valve replacement, ischemic heart disease, post-operative ischemic colitis, osteosynthesis of the right hip after a fracture, a moderate chronic renal failure and a prostatic adenocarcinoma under hormonotherapy. He was febrile (maximum fever 39.5°C) following the diagnosis of a urinary tract infection treated with ofloxacin. Upon examination he had a painful swelling of the left ankle suggesting arthritis, and arthritis of the right knee. He had purple infiltrated plaques on the skin of his inner thigh, suggesting panniculitis, and a 3/6 systolic murmur at heart auscultation. Blood analysis showed an inflammatory response with CRP at 211 mg/L and a leukocyte count of 17,000/mm^3^ with a predominance of neutrophils, associated with acute renal failure (creatinine level of 200 μmol/L with an estimated clearance of 29 mL/min and blood urea nitrogen at 20.1 mmol/L). Ankle joint aspiration was purulent and culturing only resulted in growth of *Staphylococcus epidermidis*, which is considered a contaminant. His arthritis was first treated as a microcrystalline arthropathy using colchicine with no improvement. After 3 days, he became feverish again, with no chills. Blood cultures were positive after 5 days of growth and showed small Gram-negative helical-shaped rods, suggestive of the *Campylobacter* genus. Bacterial colonies were obtained in trypcase soy agar (Difco, Becton Dickinson) enriched with 5% horse blood for 2–3 days at 37°C or 2 days at 42° under microaerophilic conditions (Figure 1). The patient was prescribed with piperacillin-tazobactam and ciprofloxacin for 4 weeks (potential endocarditis and the polyarthritis). The patient quickly showed clinical and general improvement. Antibiotics were stopped after 3 weeks because of an agranulocytosis, but by that time, the patient was clinically cured of this infection. MALDI-TOF MS performed in the context of routine analysis failed to identify the isolate and showed poor scores (unacceptable score for identification of 1.1 with *H. pullorum* with the Bruker Daltonics Biotyper). Profiles obtained with six different colonies from the isolation plate were superimposed and showed a unique profile (Supplemental Figure S1A). The two highest scores obtained were *Staphylococcus capitis* and *Lactobacillus paracasei;* these profiles were then extracted from the database and compared with that of strain S15. However, as shown in Supplemental Figures S1B,C, these two profiles were totally different from strain S15, suggesting distant organisms not phylogenetically related.

**Figure 2:**
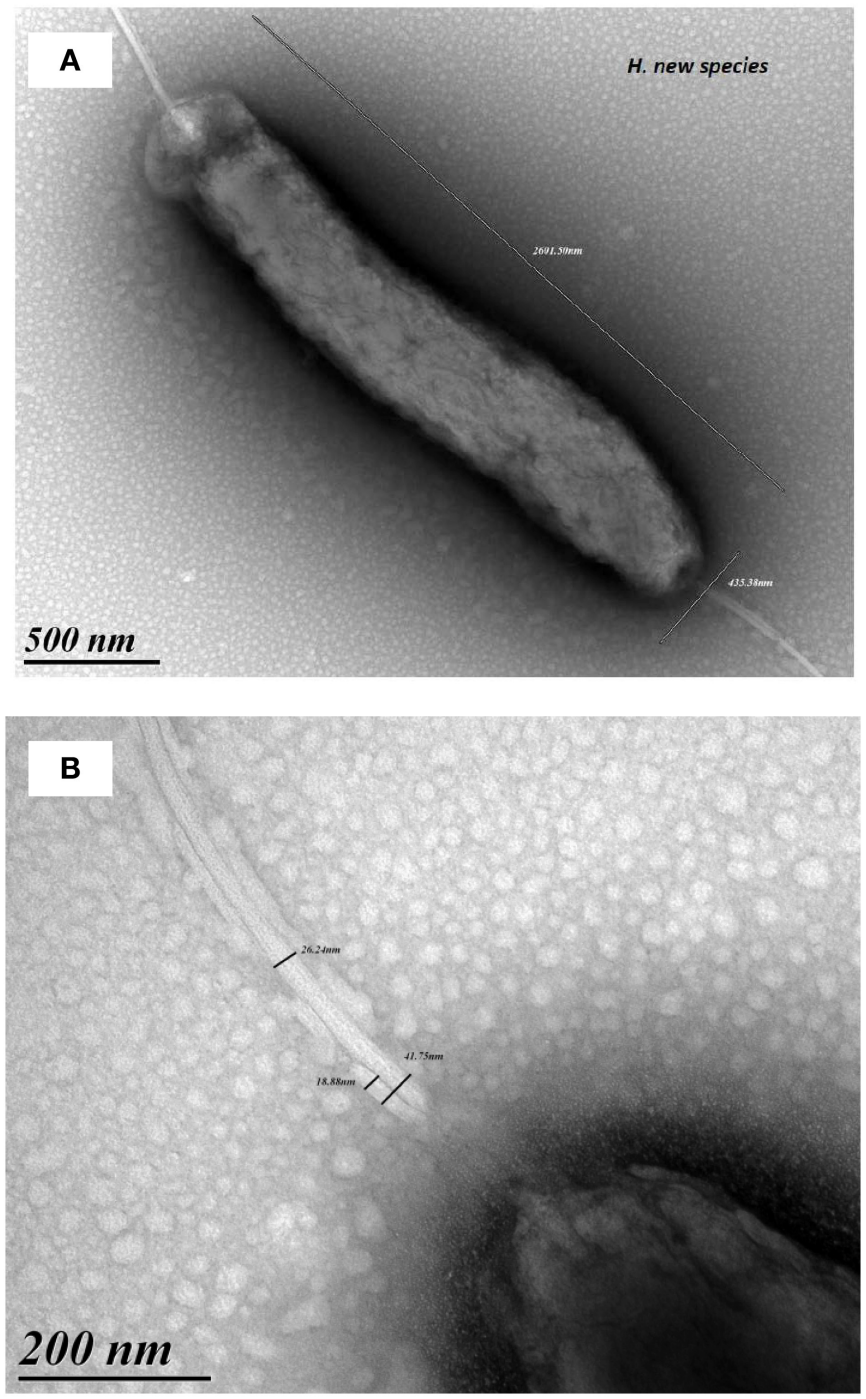
Electron microscopy pictures of strain S15. Microscopic examinations revealed a rod-shaped bacterium 3 μm in length and 0.5 μm width **(A)**. Pictures show amphitrichous sheathed flagella with a diameter around 60 nm **(B)**.

Bacterial cells were motile, curved and Gram-negative, evoking a *Campylobacter*. Fuzzy colonies were visible on trypcase soy agar plates, at 37 and 42°C after a 48 h incubation under microaerophilic conditions. There was no visible growth in a CO_2_ enriched or anaerobic atmosphere. Bacterial cells underwent transformation to coccoidal forms upon exposure to air and in cultures after prolonged incubation. Tests performed on the colonies revealed the presence of catalase and oxidase activities. Gallery API Campy showed that the isolate was positive for nitrate reductase, esterase, and alkaline phosphatase. The isolate also reduced triphenyl-tetrazolium chloride. There was no production of H_2_S, nor any detectable activity of urease, γ -glutamyl transpeptidase, pyrrolidonyl arylamidase, L-arginine arylamidase, L-aspartate arylamidase, or hippuricase. Glucose was also not assimilated (data not shown). Microscopic examinations revealed a rod-shaped bacterium with a size of 3 μm in length and 0.5 μm in width (Figure 2A). Two amphitrichous sheathed flagella with a diameter around 60 nm (Figure 2B) were visible. According to antibiotic sensitivity testing, the strain was susceptible to the β-lactams, ampicillin and amoxicillin-clavulanate, but resistant to cephalothin (diameter: 6 mm), gentamicin, erythromycin and tetracycline. It was also resistant to ciprofloxacin (MIC at 2 μg/mL) and intermediate to nalidixic acid (diameter: 12 mm).

### Genomic content

The genome of the S15 isolate was assembled into 43 contigs, with an N50 of 102,565 bp and the largest contig being 214,427 bp. The total size of the genome was evaluated around 1,708,265 base-pairs and contained 1,697 coding sequences, two copies of rRNA genes, three putative miscellaneous RNAs, 1 tmRNA, and 38 tRNAs, for a total of 1,741 genes. The GC content of S15 is 37.80%. Among the genes homologous to previously described sequences, we identified one putative toxin-/anti-toxin system, similar to YwqK found in *H. bilis*. Regarding resistance genes, we detected at least 4 multidrug resistance loci (AcrB and SecD protein sequences were 69–70% identical to *Helicobacter himalayensis* proteins; a MATE-family multidrug export protein showing 61% similarity with a similar system identified in *H. himalayensis*, and a system that showed 67% homology with QacE of *Campylobacter helveticus*; and one locus encoding heavy metal resistance). Other putative genes involved in virulence were a type II secretion system showing 50% similarity with the Eps system of *H. hepaticus* and a type VI secretion system IcmF identified in *Helicobacter trogontum* and *H. bilis* that is involved in survival within macrophages. Analysis of genome content confirmed enzyme activities for catalase, thiol-oxidase, and esterase and phosphatases but failed to identify genes encoding urease activity. Overall, 30% of CDSs (*n* = 509) detected in the genome were classified as hypothetical proteins. Comparison of important house-keeping genes (ATPase subunits, DNA polymerase, *pur*A, *gro* genes) showed maximum similarity for protein sequences between 60 and 91%. The predicted protein sequence of GyrA contained an isoleucine at amino acid position 81 known to contribute to the quinolone resistance phenotype in *H. pylori*; this confirms the *in vitro* ciprofloxacin resistance result described earlier. The flagellar operon contained 32 ORFs showing limited similarity to all publicly available sequences, between 25 and 65% for FliK and FliL, respectively. S15 does not encode homologs of CDT and γ-glutamyltranspeptidase, the main toxins of the *Helicobacter* genus.

### Phylogenetic analysis

**Figure 3:**
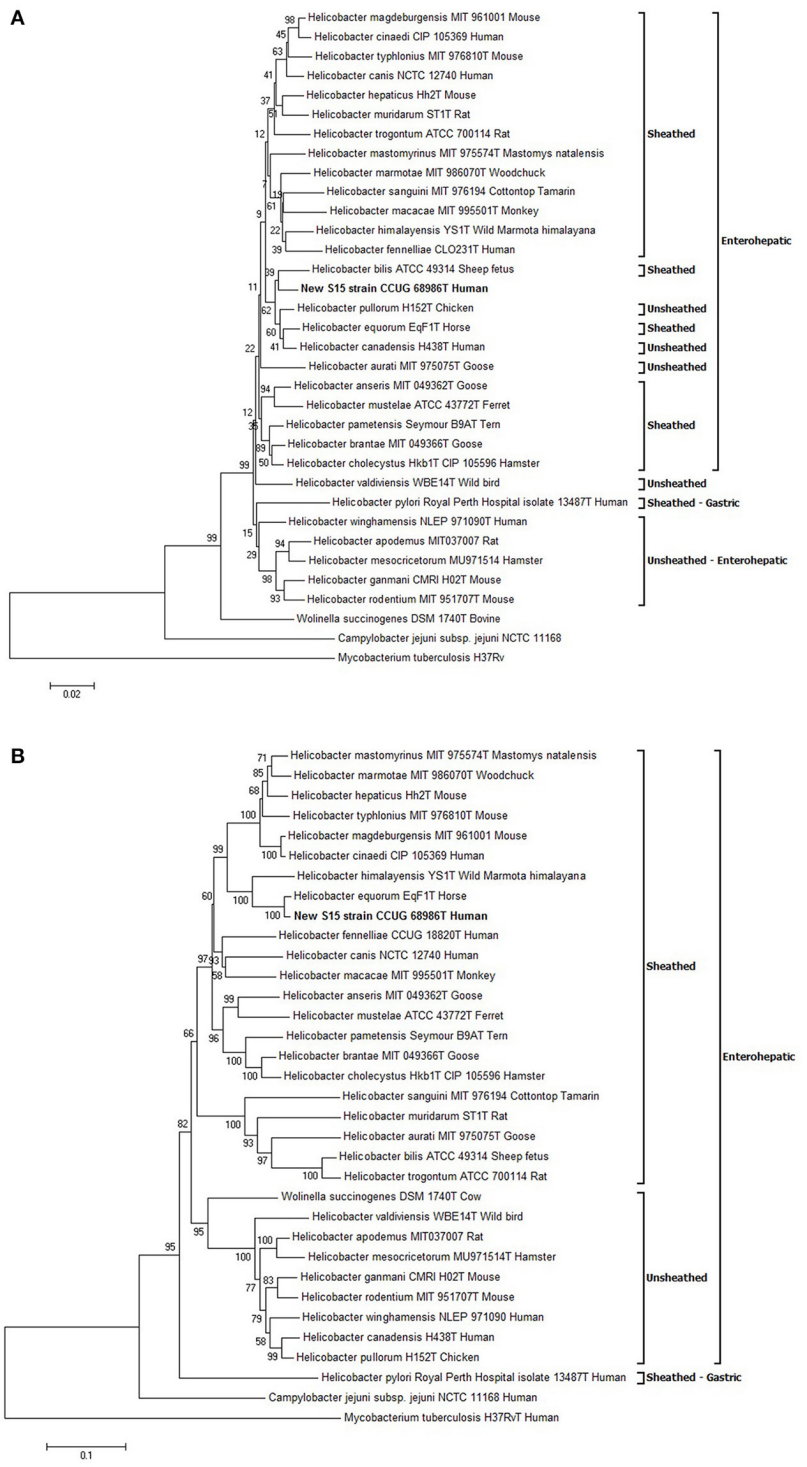
Phylogenetic trees. Evolutionary relationships of *Helicobacter* taxa based on the *16S rRNA* gene **(A)** and GyrA protein encoded gene **(B)**. The phylogeny presented is based on the alignment of approximately 1,400 nucleotides of the 16S rRNA gene and 700 residues of the GyrA protein. The phylogenetic analyses were generated with the neighbor-joining method. The percentage of replicate trees in which the associated taxa clustered together in the bootstrap test (1,000 replicates) is shown next to the branches. The trees are not rooted and drawn to scale, with branch lengths in the same units as those of the evolutionary distances used to infer the phylogenetic tree. The evolutionary distances were computed using the Kimura 2-parameter method for *16S rRNA* gene, the Poisson correction method for GyrA and are represented in the units of the number of base substitutions per site. The analysis included 34 sequences. All ambiguous positions were removed for each sequence pair. Evolutionary analyses were conducted using MEGA6. All sequences are labeled according to species, strain name, collection number in brackets*. Mycobacterium tuberculosis* was used as the outgroup sequence. All accession numbers are provided in Supplementary Table S2.

The sequences of several genes (16S rRNA gene, *hsp60, gyrA* and *gyrB*) were compared between strain S15 and closely related *Helicobacter* spp.*;* sequence identities are shown in Table 1. Based on these data, the two most genetically similar species were *H. pullorum* for the *16S rRNA* gene and *H. equorum* for the three other genes. Phylogenetic trees were constructed from nucleotide (16S rRNA gene, *hsp60* and *gyr*B genes) or predicted amino acid alignments (for GyrA). The sequences obtained by Ménard et al., were used for all *Helicobacter* spp. except for the S15 sequences, which were obtained from genome sequencing (Ménard et al., 2016). The tree obtained with the 16S rRNA gene showed that S15 clustered in a subgroup of enterohepatic *Helicobacter* species close to *H. pullorum* (Figure 3A). The phylogenetic position of S15 was then assessed using the *hsp*60 (Supplemental Figure S2A) and *gyr*B nucleotide sequences (Supplemental Figure S2B), and GyrA protein sequence (Figure 3B), a relevant marker for determining Helicobacter relatedness. In our case, the *rRNA 16S* sequence of strain S15 was 97% with *H. equorum* and only 96% with the sequence of *H. bilis*. Information extracted from these trees suggests that the S15 strain most likely corresponds to a new enterohepatic sheathed *Helicobacter* species close to *H. equorum*. The closest relatives of S15, identified using the phylogenetic trees, were used to calculate *in silico* values of DDH and ANI. Pairwise ANI-values of the closest strains identified by the algorithm in the JSpeciesWS database are depicted in Supplementary Tables S3A,B, respectively. The highest DDH-values were 39.40% obtained for the comparison with the genome of *H. canadensis* MIT98-5491 and 26.5% for *H. equorum* QF1. Comparison with the other species from the *16s RNA* and GyrA phylogenetic trees (*H. bilis* and *H. pullorum*) yields a distance of ≥0.975 and a probability that DDH is >70% between these species and S15 of 0%. The highest ANI-value was 93% between S15 strain and *H. equorum* QF1. A comparison with similarly distant species was around 70% concerning *H. canadensis* MIT98-5491 and *H. himalayensis* YS1, thus below the cut-off of 95%.

**Table 1:** Best BLAST hits for the target genes used in the present study.

**Target**	**Best match–description**	**Query cover (%)**	***E*-value**	**Identity (%)**	**Accession no**.
*16S* rRNA gene	*Helicobacter pullorum* strain ATCC 51801 16S ribosomal RNA gene, partial sequence	99	0.0	98	NR 043053.1
*hsp60 gene*	*Helicobacter equorum* strain EqF2 *hsp60* gene, partial cds	63	0.0	95	DQ888714.1
*gyrB gene*	*Helicobacter equorum* strain EqF1T *gyrase B* gene, complete cds	100	0.0	96	KM668548.1
*gyrA gene*	*Helicobacter equorum* strain EqF1T DNA *gyrase A* gene, partial cds	85	0.0	95	KM492934.1

## Discussion and conclusion

We identified in the blood cultures from a febrile patient a Gram-negative rod-shaped mobile bacterium requiring a prolonged time/incubation (almost 5 days) for detectable growth. MALDI-TOF MS used in the routine identification process failed to reliably identify this bacterium (named S15 strain), because of the bacterial protein spectrum absent in the current database. Its belonging to the *Helicobacter* genus was suggested by the phenotypic characteristics of the strain.

The whole genome sequencing data strongly provided evidence for a strain close to, but distinct from, several *Helicobacter* species. A clear distinction can be made between S15 and the major well-characterized *Helicobacter* species based on the phenotypic characteristics (e.g., ability to grow at 42°C, alkaline phosphate hydrolysis, number and disposition of flagella; Supplementary Table S3). WGS analysis provided evidence that the S15 strain is close to *H. equorum*, a bacterium that does not commonly infect humans (Moyaert et al., 2009) and is rarely found in human samples. It should be noted that the number of nucleotide sequences in public databases has evolved rapidly facilitating genome comparison. Public databases already contain an impressive number of *16S rRNA* gene sequences, a lower number of *gyrA/B* sequences and only a limited diversity of *Helicobacter* genomes. Based on all available sequences, comparison at the whole genome level showed a sharp distance between our S15 strain and *H. equorum* but clearly suggest that our strain belongs to a likely novel *Helicobacter* species. We propose to associate the present strain with the Latin name of the place of isolation; Caesarodunum (Tours, France) and suggest “*Helicobacter caesarodunensis*” for further description of this new bacterium.

Septicemia associated with a *Helicobacter* that is not characterized at the species level is not scarce (Schwarze-Zander et al., 2010). As soon as the databases associated with MALDI-TOF MS systems are updated with the peptide mass spectra of strain S15, the identification of S15-like Helicobacters will be improved in routine clinical laboratory settings. Further isolations of this bacterium in patient clinics will enable a better understanding of bacterial pathogenicity, origin, and potential routes of transmission.

## Genome sequence accession

The whole genome sequence of the S15 isolate has been deposited at DDBJ/ENA/GenBank under the accession number NESU00000000. The version described in this paper is version NESU01000000. The *16S rRNA* gene has been also deposited under the accession number KX057485.

## Ethics statement

Considering that the infection was not trivial, the patient was informed that specific investigations will be needed as the organism responsible for infection was not common. The patient gave oral consent and the strain identified in the blood cultures was shipped to the National Reference Center for Campylobacters and Helicobacters following further macroscopic visualization on blood agar. The current study was carried out in accordance with national and local recommendations. The patient gave his consent to publish the report.

## Author contributions

NvdM-M was involved in strain isolation, maintenance, and manuscript writing. LuB: performed the phenotypic experiments, analyzed the data, and wrote the manuscript. SD: performed genome assembly, annotation, and submission of the whole genome sequence to the NCBI database. AL and LoB: contributed to the clinical follow-up of the patient. NG, AS, and FH: were involved in sequence and genomic analyses. AC: performed MALDI-TOF MS analysis, protein profile comparisons and was involved in strain preservation and cultivation for culture collections. AS and FH: contributed to the sequencing and assembly of the *H. equorum* genome used for ANI and DDH. AD, SL, and EG: performed strain characterization experiments. FM was involved in writing the manuscript. AG: contributed to manuscript writing and correction and performed ANI and DDH determinations. PL: designed the experiments, analyzed the data, and wrote the manuscript. PF: involved in the analysis of genome annotation and writing the manuscript.

### Conflict of interest statement

The authors declare that the research was conducted in the absence of any commercial or financial relationships that could be construed as a potential conflict of interest.
