# Supplementary material for: A Potential New Human Pathogen Belonging to Helicobacter Genus, Identified in a Bloodstream Infection
Source: Front Microbiol. 2017 Dec 18;8:2533. doi: 10.3389/fmicb.2017.02533 (PMC5741639; doi:10.3389/fmicb.2017.02533)
Supplement: Supplementary file 1 [file Table1.DOCX]

**Supplementary Table S1. Phenotypic characteristics that differentiate *Helicobacter* S15 from other *Helicobacter* species**

| **Characteristics** | **1** | **2** | **3** | **4** | **5** | **6** | **7** | **8** | **9** | **10** | **11** | **12** | **13** | **14** | **15** | **16** | **17** | **18** | **19** | **20** | **21** | **22** | **23** | **24** | **25** | **26** |
| --- | --- | --- | --- | --- | --- | --- | --- | --- | --- | --- | --- | --- | --- | --- | --- | --- | --- | --- | --- | --- | --- | --- | --- | --- | --- | --- |
| **Catalase production** | + | + | + | + | + | + | + | (+) | (+) | + | + | + | + | + | (+) | + | + | + | + | + | + | + | (-) | + | + | + |
| **Nitrate reduction** | + | + | + | v | - | + | + | - | + | - | - | - | - | + | + | + | + | + | + | + | + | (+) | + | - | v | + |
| **Urease** | - | - | - | - | + | + | (+) | - | - | + | + | + | - | + | - | + | (+) | + | - | - | + | - | - | + | - | + |
| **Alkalinephosphate hydrolysis** | + | + | - | - | + | + | v | (-) | (-) | + | + | + | + | - | (+) | - | v | - | + | - | v | + | - | - | - | + |
| **Gamma-glutamyl transpeptidase** | - | - | - | - | + | + | + | - | - | ND | + | + | + | - | - | + | + | + | - | - | + | - | ND | + | - | + |
| **Indoxyl acetate hydrolysis** | ND | - | - | + | (-) | + | (-) | + | (-) | - | - | (-) | + | + | - | - | (-) | - | - | - | (-) | ND | - | + | - | - |
| **Growth at 42°C** | + | - | + | + | (-) | v | v | (-) | v | + | - | (-) | + | - | + | + | v | + | + | + | - | + | - | + | + | - |
| **Growth on 1% glycine** | ND | - | - | + | - | - | - | - | - | - | - | - | - | + | V | + | (-) | ND | + | + | - | - | - | - | + | - |
| **Susceptibility to:** |  |  |  |  |  |  |  |  |  |  |  |  |  |  |  |  |  |  |  |  |  |  |  |  |  |  |
| **Nalidixic acid (30µg)** | I | R | R | R | R | S | R | S | S | R | R | R | S | R | S | R | R | R | I | R | R | S | S | S | S | ND |
| **Cephalotin (30µg)** | R | R | S | R | S | R | S | S | I | S | R | S | I | R | S | R | S | R | R | R | S | R | R | R | R | ND |
| **Periplasmic fibril** | - | - | - | - | - | - | + | - | - | - | + | - | - | - | + | - | - | + | - | - | - | - | - | + | - | + |
| **No. flagella per cell** | 2 | 1 | 1 | 1-2 | 4-8 | 4-8 | 14-20 | 2 | 1-2 | 4-8 | 10-14 | 2-5 | 2 | 2 | 2 | 3-14 | 10-20 | 5-7 | 1 | 2 | 10-23 | 2 | 2 | 7-10 | 2 | 6-12 |
| **Sheated flagella** | Yes | Yes | No | No | Yes | Yes | Yes | Yes | Yes | Yes | Yes | Yes | Yes | Yes | Yes | Yes | Yes | Yes | Yes | No | Yes | No | No | Yes | Yes | Yes |
| **Distribution of flagella** | A | M | M | B | B | P | B | B | B | B | B | B | B | B | B | B | B | B |  | B | B | B | B | B | B | B |

| Taxa: 1, New S15 strain; 2, *H. equorum*; 3, *H. pullorum*; 4, *H. canadensis*; 5, *H. pylori*; 6, *H. mustelae*; 7, *H. felis*; 8, *H. fennelliae*; 9, *H. cinaedi*; 10, H. nemestrinae; 11, *H. muridarum*; 12, *H. acinonychis*; 13, *H. canis*; 14, *H. hepaticus*; 15, *H. pametensis*; 16, *H. bilis*; 17, *H. bizzozeronii*; 18, *H. trogontum*; 19, *H. cholecystus*; 20, *H. rodentium*; 21, *H. salomonis*; 22, *H. mesocricetorum*; 23, *H. ganmani*; 24, *H. aurati*; 25, *H. typhlonius*; 26, *H. cynogastricus*. |
| --- |
| +, 100% strains positive; -, 100% strains negative; (+), 80-94% strains positive; v, 42-66% strains positive; (-), 7-33% strains positive; S, sensitive; R, resistant; I, intermediate; ND, not determined; A, Amphitrichous; B, Bipolar; M, Monopolar; P, peritrichous. |
| Data were obtained from study published by Moyaert and colleagues [20] except for *Helicobacter* S15 strain (this study). |

**Supplementary Table S2. . Strains included in this phylogenetic analyses**

| **Species** | **Host, site, pathology, flagella morphology** | **Type strain** | **Name and collections** | Genbank accession numbers | | | | gyrA G+C % |
| --- | --- | --- | --- | --- | --- | --- | --- | --- |
|  |  |  |  | ***16S rDNA*** | hsp60 groEL | gyrB | gyrA |  |
| *Campylobacter jejuni* subsp. *jejuni* | Human, stool, campylobacteriosis (United Kingdom, 1977), unsheathed | no | **NCTC 11168,** ATCC 700819 | AL111168 | AL111168 | AL111168 | AL111168 | 33.5 |
| *H. anseris* | Goose, feces (Boston, MA, USA), sheathed | type | ATCC BAA-1299, **CCUG 52421**, **MIT 04-9362** | DQ415545.1 | KT198998 | KT198999 | KT599911 | 35.8 |
| ‘*H. apodemus*’ | Rat, feces (USA) | NV | MIT-03-7007 | JRPC01000143.1 | JRPC01000018.1 | NZ_JRPC01000029 | JRPC01000004.1 | 36.3 |
| *H. aurati* | Goose, feces (Boston, MA, USA), sheathed | type | 97-5075C, ATCC BAA-1, **CCUG 47791**, **MIT 97-5075**, MIT 97-5075c | NR_025124.1 | KT697621 | AJ969179.1 | KT697620 | 38.4 |
| *H. bilis* | Aborted sheep fetus, liver and fluids (Brookings, SD, USA), sheathed | no | **ATCC 49314** | JRPI01000001.1 | JRPI01000001.1 | JRPI01000003.1 | JRPI01000002.1 | 39.2 |
| *H. brantae* | Goose, feces (Boston, MA, USA), sheathed | type | ATCC BAA-1298, **CCUG 52420**, **MIT 04-9366** | NR_043799.1 | KT697623 | KT198997 | KT697622 | 40.7 |
| *H. canadensis* | Human, stool, gastroenteritis (Canada), unsheathed | type | **H438,** 98-5491, ATCC 700968, **CCUG 47163, NLEP-16143, NCTC 13241, MIT 98-5491** | ABQS01000108 | ACSF01000002 | ACSF01000001 | ACSF01000002 | 35.2 |
| *H. canis* | Human (51/2-year-old boy), stool, gastroenteritis, sheathed | no | **NCTC 12740,** A805/92, E554-91 | AZJJ01000002 | AZJJ01000001 | AZJJ01000001 | AZJJ01000002 | 46 |
| *H. cholecystus* | Hamster, gallbladder, cholangiofibrosis, sheathed | type | **ATCC 700242,** [**CIP 105596**](http://www.straininfo.net/strains/151656/browser)**, Hkb-1** | NR_043108 | KM668522 | KM507019 | KM065461 | 36.8 |
| *H. cinaedi* | Human, blood (42 year-old woman), gynaecological case, sheathed | no | CCUG 15432, **CIP 105369,** LMG 8559, Skövde B1205 | KJ534298 | KM668523 | KM668545 | KM023718 | 40.1 |
| *H. equorum*  ^#^ | Horse, feces (Ghent, Belgium), sheathed | type | EqF1, LMG 23362, CCUG 52199 | KJ534299 | KM492933 | KM668548 | KM492934 | 40 |
| *H. fennelliae* | Aboriginal child (14-month-old), blood, bacteremia (with vomiting, diarrhea, fever, and dry cough symptoms), sheathed | type | 231, ATCC 35684, **CCUG 18820,** CIP 103758, **DSM 7491**, DSM 7591, Fennel CLO-2, Fennell 231, LMG 13645, LMG 18294, LMG 7546, NCTC 11612, strain CLO-231 | M88154 | KM668526 | AB595161.1 | KM492935 | 43.2 |
| *H. ganmani* | Mouse, intestine (laboratory mice), unsheathed | type | [**CIP 106846**](http://www.straininfo.net/strains/302662)**,** [**CMRI H02**](http://www.straininfo.net/strains/302663)**,** [M2](http://www.straininfo.net/strains/328717) | AF000221 | KM668528 | KM507021 | KM065462 | 41 |
| *H. hepaticus* | Mouse (NCID/NCr), liver, active, chronic hepatitis, sheathed | type | **Hh-2, ATCC 51448,** BCRC 17336, CCRC 17336, **CCUG 33637, CIP 104100,** MIT Hh-2, LMG 16316 | U07574 | AY787943 | AJ969197 | KM023719 | 38 |
| *H. himalayensis* | Wild *Marmota himalayana*, gastric mucosa, sheathed | type | YS1, CGMCC 1.12864, **DSM28742** | KJ716794.1 | KJ716793.1 | KJ716792.1 | /^#^ | 44.1 |
| ‘*H. magdeburgensis*’ | Mouse, cecum (USA), sheathed | NV | MIT 96-1001 | JRPE01000101.1 | JRPE01000070.1 | JRPE01000056.1 | JRPE01000123.1 JRPE01000034.1 | 41.7 |
| *H. macacae* | Rhesus monkey, intestinal tract, chronic idiopathic colitis, sheathed | type | CCUG 55313, LMG 26486, **MIT 99-5501** | AZJI01000005 | AZJI01000009 | AZJI01000001.1 | AZJI01000005 | 43.1 |
| *H. marmotae* * | Necropsy of wild-caught adult woodchuck (Marmota monax), liver, sheathed | type | ATCC BAA-546, CCUG 52419, **MIT 98-6070** | NR_041825 | KM668529 | AB595164 | KM492936 | 39.3 |
| *H. mastomyrinus* | Mastomys natalensis (African rodent), sheathed | type | ATCC BAA-1046, **CCUG 52417**, **MIT 97-5574** | NR_115314.1 | KT198995 | AB595162.1 | KT198996 | 41.6 |
| *H. mesocricetorum* * | Asymptomatic Syrian hamsters (*Mesocricetus auratus*), feces, unsheathed | type | **ATCC 700932,** CCUG 45420, **MU 97-1514,** MU1514 | AF072471 | KM668525 | AB595163 | KM065464 | 36.2 |
| *H. muridarum* | Rat, intestinal mucosa (ileum) (Australia), sheathed | type | **ST1, ATCC 49282, CCUG 29262, CIP 104248,** Lee ST1, LMG 12685, LMG 13646, **NCTC 12714** | M80205 | AY787951 | AJ969198.2 | KM065465 | 36.4 |
| *H. mustelae* | Ferret (*Mustela putorius furo*), gastric mucosa (NY, USA), gastric ulcer, sheathed | type | **ATCC 43772, CCUG 25715,** CIP 103759, Fox R85-136P, LMG 13647, LMG 18044, LMG 8616, **NCTC 12198,** R-1877, R-85-13 6P, **R-85-13-6,** R-85-136P | FN555004 | FN555004 | FN555004 | FN555004 | 45.7 |
| *H. pametensis* | Tern, fecal sample, sheathed | type | **Seymour B9A, ATCC 51478,** B9, CCUG 29255, CCUG 34905, **CIP 104249,** LMG 12678, NCTC 12887, **Seymour B9** | JADE01000006 | JADE01000008 | JADE01000009 | JADE01000007 | 42.1 |
| *H. pullorum* | Asymptomatic broiler chicken at slaughter (Switzerland), unsheathed | type | **H152,** ATCC 51801, Burnens H152, **CCUG 33837, CIP 104787,** LMG 16317, **NCTC 12824** | NR_043053 (ex. L36141) | DQ059426 | KM668544 | DQ836338 | 34.9 |
| *H. pylori* | Human gastric antrum (Australia), sheathed | type | **ATCC 43504**, BCRC 15415, CCRC 15415, CCUG 15815, CCUG 15815 A, CCUG 15815 B, **CCUG 17874**, CCUG 39500, CCUG 41351, CIP 103995, DSM 4867, JCM 12093, JCM 7653, K0045659, KCTC 12083, KCTC 2948, LMG 18041, LMG 18045, LMG 19449, LMG 7539, LMG 7540, **NCTC 11637**, strain Royal Perth Hospital 13487, R-1876, **Royal Perth Hospital 13487**, RPH 13487, strain Royal Perth Hospital 13487, VTT E-021812 T | AIHX01000070.1 | AIHX01000147.1 | AIHX01000050.1 | KT198991 | 42.4 |
| *H. rodentium* * | Laboratory mouse (C57bl/6x129), feces, unsheathed | type | **MIT 95-1707, ATCC 700285,** R-5081 | U96296 | JHWC01000001 | JHWC01000003 | JHWC01000002 | 42 |
| ‘*H. sanguini*’ | Cotton top tamarin, fecal sample, sheathed | NV | MIT 97-6194 | JRMP01000157.1 | JRMP01000178.1 | JRMP01000172.1 | JRMP01000178.1 | 40.2 |
| *H. trogontum* | Rat, intestinal mucosa (Brazil), sheathed | type | **ATCC 700114, CCUG 49050**, LRB 8581 | JRPL01000013.1 | JRPL01000001.1 | NZ_JRPL01000004.1 | JRPL01000001.1 | 36.1 |
| [*H. typhlonius*](http://www.straininfo.net/taxa/search?q=helicobacter+typhlonius) | Mouse (IL-10^_/_^ knockout on a C57BL6/129-Ola background) with rectal prolapse (USA), sheathed | type | ATCC BAA-367, **CCUG 48335, CIP 107729, MIT 97-6810** | AF127912 | KM668537 | AB595165 | KM065469 | 41.1 |
| *H. valdiviensis* | Wild bird, faecal samples (Valdivia, Southern Chile), unsheathed | type | **WBE14**, **CECT 45 8410**, LMG 27920. | KF549903.1 | KF549905.1 | KT599909 | KT590048 | 35.7 |
| ‘*H. winghamensis*’ | Human (male 2 yrs), stool, symptoms of gastroenteritis (Wingham, Ontario, Canada, 1997), unsheathed | NV | **NLEP 97-1090, ATCC BAA-430** | ACDO01000013 | ACDO01000019 | ACDO01000012 | ACDO01000016 | 37.7 |
| *Wolinella succinogenes* | Bovine rumen, unsheathed | type | Wolin, ATCC 29543, CCUG 12550, CCUG 13145, CIP 103760, **DSM 1740,** FDC 602W, KCTC 5018, LMG 7139, LMG 7463, LMG 7466, LMG 7608, NCTC 11488 | BX571657 | BX571657 | BX571658 | BX571658 | 50.9 |
| *Mycobacterium tuberculosis* | Human, lung, pulmonary tuberculosis, no flagella | type | H37Rv, ATCC 25618, ATCC 27294, ATCC 9360, CCUG 37357, CCUG 38148, CIP 104475, CIP 64.31, CNCTC 7301, CNCTC My 331/88, G.Kubica TMC 102, KPM T021, MG 1, NCTC 13144, NCTC 7416, TMC 102 | AL123456 | AL123456 | AL123456 | AL123456 | 63.9 |

Data adapted from Ménard A *et al.* [19]

**Supplementary Table S3A. Result of Average Nucleotide Identity obtained for the closest sequenced organisms**

|  | ***H. pullorum* 229313 12.** | ***H. bilis* AAQJH.** | ***H. himalayensis* YS1.** | ***H. cinaedi* CCUG 18818.** | ***H. felis* ATCC 49179.** | ***H. canadensis* MIT 98-5491.** | ***H. mustelae* 12198.** | ***H. hepaticus* ATCC 51449.** | ***H. equorum* QF1.** | **New strain*.* S15** |
| --- | --- | --- | --- | --- | --- | --- | --- | --- | --- | --- |
| ***H. pullorum* 229313** |  | 67.47 [20.40] | 67.60 [22.92] | 67.75 [25.56] | 65.53 [16.23] | 78.57 [67.00] | 66.41 [21.59] | 68.07 [27.55] | 68.43 [25.19] | 68.27 [26.29] |
| ***H. bilis* AAQJH** | 67.45 [14.46] |  | 68.10 [15.52] | 73.32 [20.10] | 65.68 [11.05] | 67.64 [14.13] | 66.86 [13.61] | 68.74 [18.88] | 68.33 [15.69] | 69.06 [16.77] |
| ***H. himalayensis* YS1** | 67.99 [22.29] | 68.12 [21.70] |  | 69.84 [33.14] | 66.09 [16.33] | 67.87 [21.95] | 67.54 [20.54] | 69.42 [32.19] | 71.11 [41.49] | 71.30 [41.68] |
| ***H. cinaedi* CCUG18818** | 67.84 [21.55] | 72.95 [22.63] | 69.51 [27.59] |  | 66.08 [14.50] | 67.78 [21.85] | 67.07 [19.27] | 75.06 [49.86] | 69.18 [29.46] | 69.53 [30.99] |
| ***H. felis* ATCC49179** | 65.40 [17.49] | 65.39 [17.32] | 65.72 [18.20] | 65.66 [19.37] |  | 65.17 [17.83] | 65.96 [24.34] | 65.15 [20.88] | 65.50 [18.38] | 65.65 [19.11] |
| ***H. canadensis* MIT98-5491** | 78.71 [70.65] | 68.08 [20.70] | 68.19 [23.59] | 68.24 [27.20] | 65.96 [17.22] |  | 67.04 [22.28] | 68.50 [28.32] | 69.80 [27.68] | 70.72 [28.87] |
| ***H. mustelae* 12198.** | 66.83 [23.23] | 67.30 [21.03] | 67.49 [23.24] | 67.25 [25.66] | 66.23 [25.37] | 66.76 [23.08] |  | 67.19 [25.93] | 67.37 [24.36] | 67.48 [24.95] |
| ***H. hepaticus* ATCC 51449.fa** | 67.82 [27.59] | 68.67 [25.99] | 69.23 [33.42] | 74.95 [61.09] | 65.52 [18.43] | 68.05 [27.75] | 66.88 [23.86] |  | 69.16 [35.90] | 69.37 [37.94] |
| ***H. equorum* QF1** | 68.23 [27.36] | 68.48 [24.38] | 70.86 [47.56] | 69.33 [39.14] | 65.53 [18.50] | 69.42 [29.86] | 67.19 [24.58] | 69.25 [39.14] |  | **93.28 [84.28]** |
| **New strain*.* S15** | 68.17 [27.19] | 68.83 [24.45] | 71.33 [44.80] | 69.68 [39.44] | 65.76 [17.92] | 69.68 [29.60] | 67.14 [24.15] | 69.46 [39.62] | **93.36 [80.29]** |  |

Table indicates the ANI values (alignment percentage) and the proportion of genomes used for the pairwise comparison between brackets. In bold font pairwise comparisons of the new S15 strain with other publically available selected genomes identified from phylogenetic tree. Values in bold show the highest ANI values.

**Supplementary Table S3B. Result of Average Nucleotide Identity obtained for the closest organisms appearing in the JSpeciesWS database**

| **Species** | **Strain** | **Domain** | **Phylum** | **Class** | **Order** | **Family** | **Z-Score** |
| --- | --- | --- | --- | --- | --- | --- | --- |
| ***Helicobacter typhlonius*** | MIT 98-6810 | Bacteria | Proteobacteria | Epsilonproteobacteria | Campylobacterales | Helicobacteraceae | 0.89503 |
| ***Helicobacter sp.*** | MIT 03-1616 | Bacteria | Proteobacteria | Epsilonproteobacteria | Campylobacterales | Helicobacteraceae | 0.89428 |
| ***Helicobacter sp.*** | MIT 01-6451 | Bacteria | Proteobacteria | Epsilonproteobacteria | Campylobacterales | Helicobacteraceae | 0.89264 |
| ***Helicobacter fennelliae*** | MRY12-0050 | Bacteria | Proteobacteria | Epsilonproteobacteria | Campylobacterales | Helicobacteraceae | 0.89108 |
| ***Helicobacter sp.*** | MIT 05-5293 | Bacteria | Proteobacteria | Epsilonproteobacteria | Campylobacterales | Helicobacteraceae | 0.88371 |
| ***Helicobacter sp.*** | MIT 05-5294 | Bacteria | Proteobacteria | Epsilonproteobacteria | Campylobacterales | Helicobacteraceae | 0.87553 |
| ***Helicobacter sp.*** | MIT 03-1614 | Bacteria | Proteobacteria | Epsilonproteobacteria | Campylobacterales | Helicobacteraceae | 0.85898 |
| ***Helicobacter cinaedi*** | ATCC BAA-847 | Bacteria | Proteobacteria | Epsilonproteobacteria | Campylobacterales | Helicobacteraceae | 0.85601 |
| ***Helicobacter hepaticus*** | ATCC 51449 | Bacteria | Proteobacteria | Epsilonproteobacteria | Campylobacterales | Helicobacteraceae | 0.85508 |
| ***Helicobacter cinaedi*** | CCUG 18818 | Bacteria | Proteobacteria | Epsilonproteobacteria | Campylobacterales | Helicobacteraceae | 0.8545 |
