# Supplementary material for: A Potential New Human Pathogen Belonging to Helicobacter Genus, Identified in a Bloodstream Infection
Source: Front Microbiol. 2017 Dec 18;8:2533. doi: 10.3389/fmicb.2017.02533 (PMC5741639; doi:10.3389/fmicb.2017.02533)
Supplement: Supplementary file 2 [file Image1.PDF]

Supplementary figure S1

A

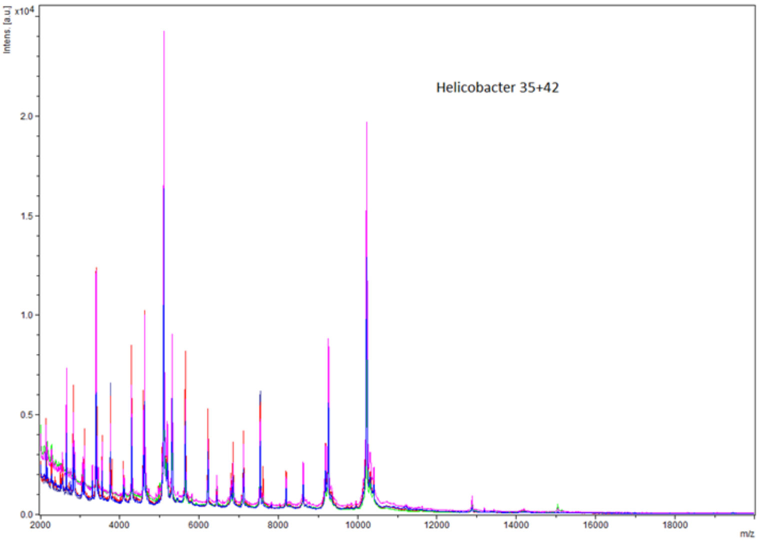

B

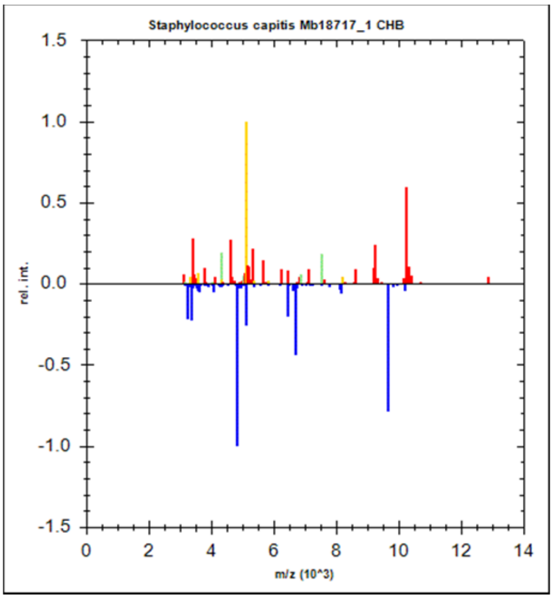

C

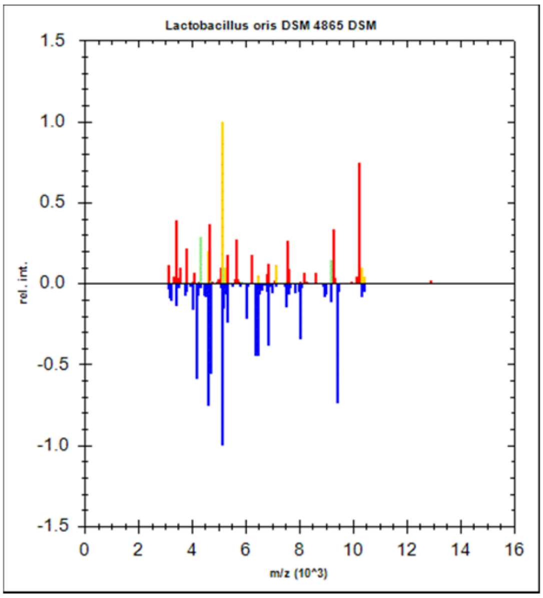

Graphical representation of classification results obtained from a match of the new strain S15 to a reference Main Spectrum Profile (MSP).

The upper half of the view displays the normalized peak list spectrum of the new S15 strain ~~*Helicobacter caesarodunum*~~. The lower half displays the peak list spectrum (displayed using an inverted intensity scale) of the MSP that is currently selected in the MSP Scores table of the Table View.

The color of each peak reflects the closeness of the match to the reference MSP (green = full match, yellow = partial match, red = no match).

MALDI-TOF performed in the context of routine analysis failed to identify the isolate and showed poor scores. Profile from different colonies showed a unique profile different from the less distant organisms detected by MALDI-TOF, *S. capitis* (B) or *Lactobacillus oris* (C).

Supplementary Figure S2A

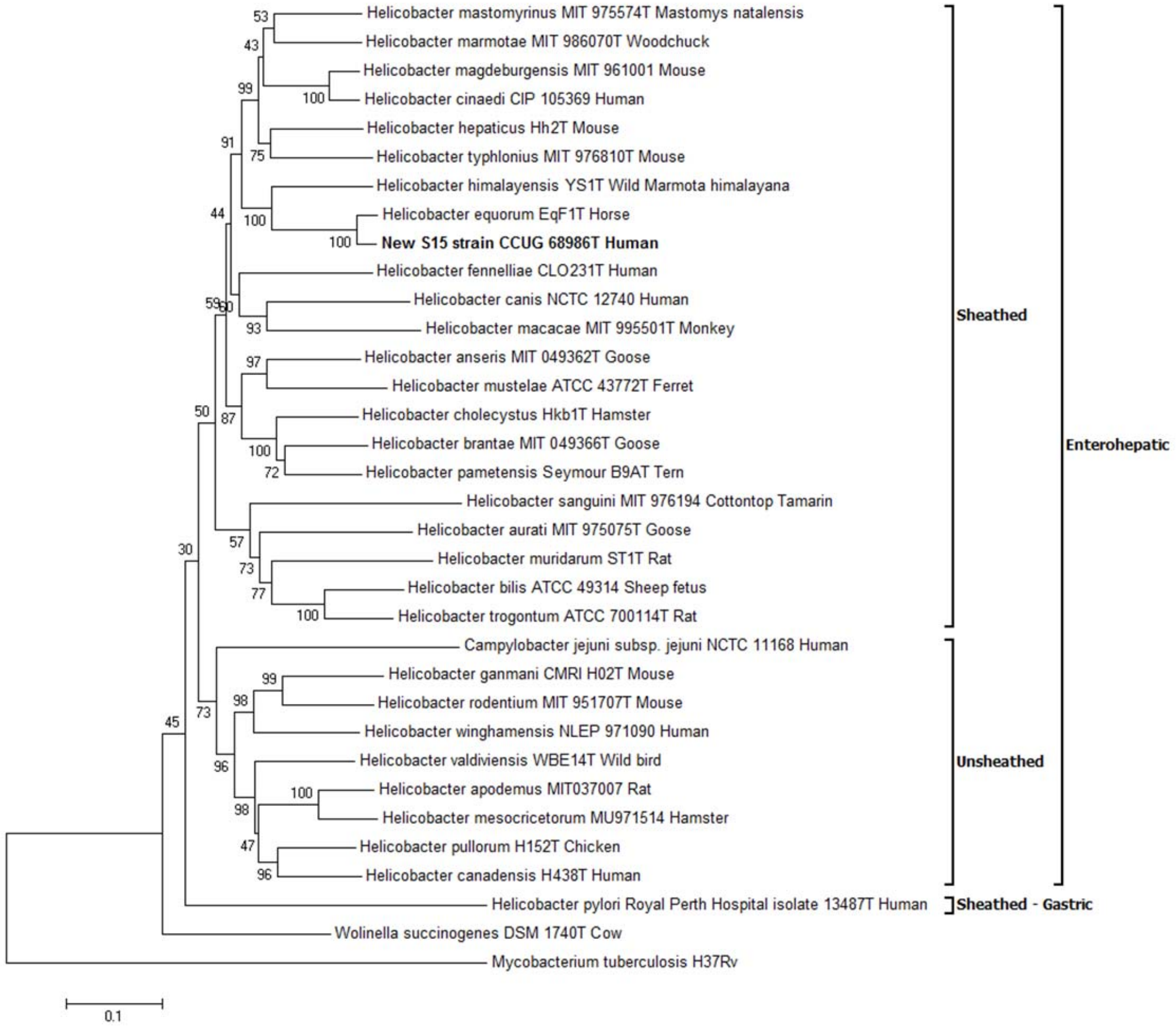

Supplementary Figure S2B

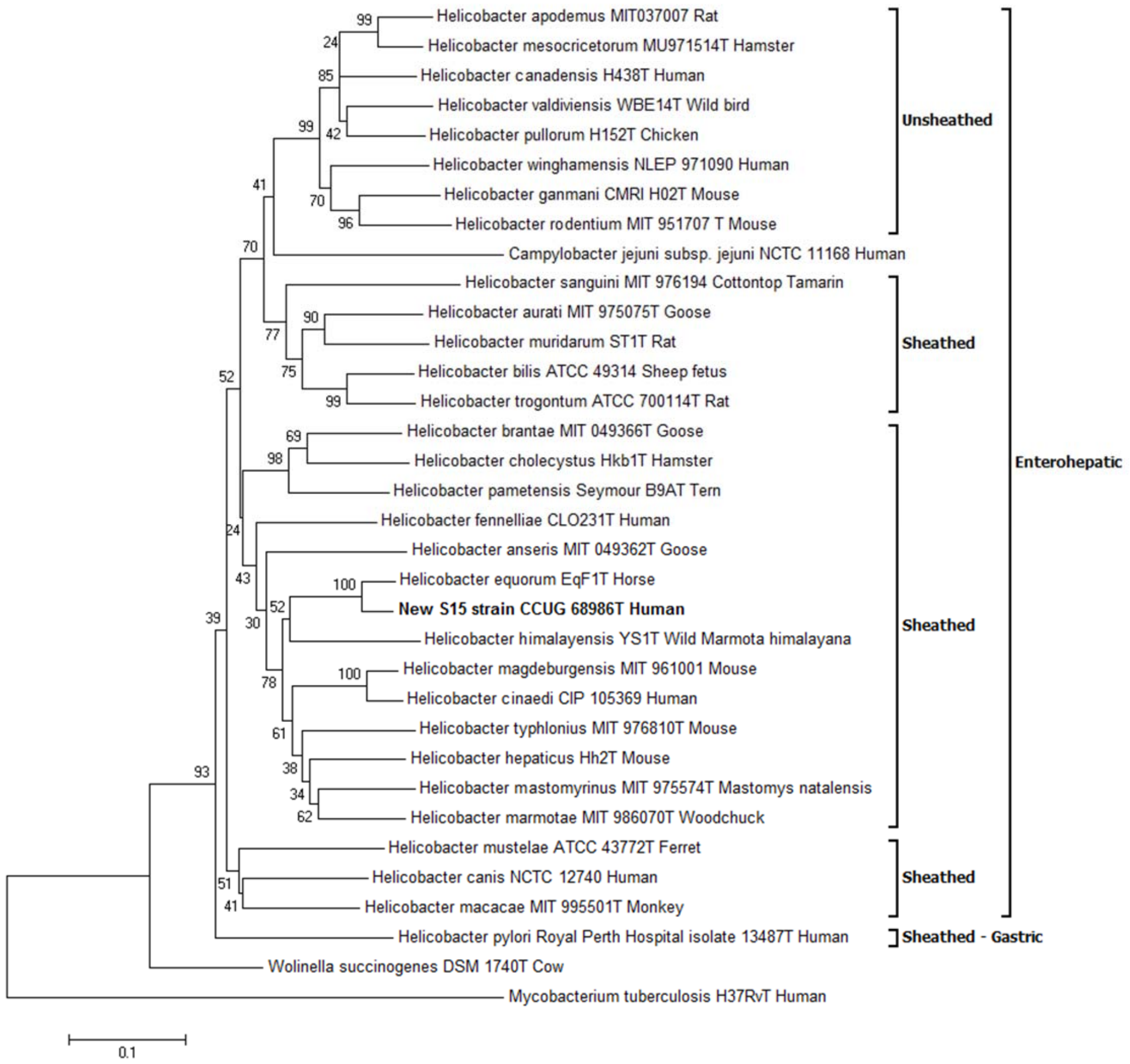

Supplementary Figure S2. *GyrB* and *hsp60* genes phylogenetic trees. Evolutionary relationships of *Helicobacter* taxa based on the *gyrB* gene (A) and *hsp60* gene (B). The phylogeny presented is based on the alignment of approximately 985 nucleotides of *gyrB* and 540 nucleotides of *hsp60*. The phylogenetic analyses were generated with the neighbor-joining method. The percentage of replicate trees in which the associated taxa clustered together in the bootstrap test (1,000 replicates) is shown next to the branches. The trees are not rooted and drawn to scale, with branch lengths in the same units as those of the evolutionary distances used to infer the phylogenetic tree. The evolutionary distances were computed using the Kimura 2-parameter method and are represented in the units of the number of base substitutions per site. The analysis included 34 sequences. All ambiguous positions were removed for each sequence pair. Evolutionary analyses were conducted using MEGA6. All sequences are labeled by species, strain name, collection number in brackets, and the species from which they were isolated. *Mycobacterium tuberculosis* was used as the outgroup sequence. All accession numbers are provided in Supplementary Table 2.
